# STRUCTURAL INEQUITIES IN OUTCOMES FOR DUAL-ELIGIBLE RESIDENTS IN ASSISTED LIVING

**DOI:** 10.1093/geroni/igac059.1654

**Published:** 2022-12-20

**Authors:** Portia Cornell, Cassandra Hua, Momotazur Rahman, Gauri Gadkari, Kali Thomas

**Affiliations:** Providence VA Medical Center / Brown University, Providence, Rhode Island, United States; Brown University School of Public Health, Providence, Rhode Island, United States; Brown University, Providence, Rhode Island, United States; Brown University School of Public Health, Providence, Rhode Island, United States; Brown University, Providence, Rhode Island, United States

## Abstract

We examined the association of AL residents’ dual-eligibility and the concentration of dually eligible residents in AL communities with residents’ risk of hospitalization and long-term nursing home admission. The exposure was dual status interacted with AL concentration: no-duals, minority-duals [<=50%] (reference group), and majority-duals [>50%]. We found that duals in AL have higher risk of hospitalization and nursing home admission than non-duals. For both duals and non-duals, moving to an AL with a high concentration of duals conferred excess risk of hospitalization. Among duals, however, lower concentration of duals in ALs increases risk of long-term nursing home admission for duals, whereas it is protective for non-duals. The association of higher hospitalization with concentration of duals suggests that quality may be a concern in communities that specialize in care for duals. However, majority-duals ALs may be better equipped to provide more comprehensive care as an alternative to nursing homes.

